# Comparison of performance with hearing aid programmed to NAL-NL1 first-fit and optimized-fit

**DOI:** 10.1590/2317-1782/20212020310

**Published:** 2021-10-18

**Authors:** Sreena Ediyarath Narayanan, Puttabasappa Manjula

**Affiliations:** 1 Department of Audiology, All India Institute of Speech and Hearing, Mysuru, Karnataka, India.

**Keywords:** Hearing Aid, NAL-NL1, First-fit, Optimized-fit, Probe-microphone Verification

## Abstract

**Purpose:**

The initial-fit provided by the hearing aid manufacturer’s software is generally a display of measurement done in the ear simulators. The need for verification of hearing aid output and gain in the real ear using probe-microphone measurement to match the prescriptive target is highlighted. The objective of the study was to evaluate the difference in real-ear aided response (REAR), real-ear insertion gain (REIG), aided thresholds, articulation index (AI) and word recognition score (WRS) in quiet, with hearing aid programmed to NAL-NL1 first-fit and NAL-NL1 optimized-fit using the probe-microphone technique.

**Methods:**

In a repeated measure experimental design, 11 participants with a mean age of 41.09 (SD=±9.95) years having moderate and moderately-severe sensorineural hearing loss were tested monaurally in two aided conditions, with a 16-channel hearing aid programmed for manufacturer’s NAL-NL1 first-fit and optimized-fit to NAL-NL1 using probe-microphone verification. The REAR, REIG, aided threshold, articulation index and word recognition scores in quiet were obtained for both aided conditions.

**Results:**

The REAR, REIG, aided threshold, AI and WRS in quiet were significantly better with the NAL-NL1 optimized-fit compared to manufacturer’s NAL-NL1 first-fit.

**Conclusion:**

The optimized-fit yields better audibility and improved word recognition in quiet. This supports best practice guidelines of many professional organizations regarding the use of probe-microphone measurement as the “Gold standard” for verification of hearing aid fitting, thereby providing better satisfaction and quality of life to hearing aid users.

## INTRODUCTION

The Independent Hearing Aid Fitting Forum (IHAFF), International Society of Audiology (ISA), American Academy of Audiology (AAA), and American Speech and Hearing Association (ASHA) consider the use of probe-microphone verification as one of the best practices for adult hearing aid fittings^(1-4)^. The probe-microphone measurement is accurate and reliable to verify the gain/output of the hearing matched to the given prescriptive formula like the National Acoustics Laboratory Nonlinear fitting procedures, NAL-NL1 and NAL-NL2 or the Desired Sensation Level input/output formula^(5)^. Though, the probe-microphone technology is available for more than three decades, a small percentage of audiologists use this to verify the hearing aid fittings^(6)^.

Many hearing aid practitioners prescribe hearing aids based on the manufacturer’s first-fit hearing aid fitting. When the first-fit is alone used, the accuracy of the target match can be low as it is carried out only in ear simulators/coupler and there is no verification of the gain of the hearing aid in real ear^(7)^. It was found that the measured 2cc coupler output of the manufacturer’s initial-fit was significantly lower than the NAL-NL1 targets by as much as 10-15 dB^(8,9)^. The real rear measurements of the initial-fit and the verified-fit from various studies were also consistent with the fact that the gain and output provided for the high frequencies are significantly lower with the initial-fit program^(10-13)^. Hearing aid with initial-fit led to reduced speech recognition and subjective satisfaction. When fitted with programmed-fit to NAL-NL2 targets a significant improvement was observed in speech intelligibility index, speech recognition in quiet and noise, and self-perceived benefit^(14-17)^.

Those studies compared the benefit with manufacturer’s proprietary first-fit and programed-fit to a given generic prescriptive target. Valente et al.^(17)^ suggested that the NAL-NL2 first-fit would have used instead of manufacturer’s proprietary first-fit, the difference in audibility would have been present but not as poor as manufacturer’s proprietary first-fit. It probes further to investigate the difference in performance with first-fit of a given generic prescriptive targets and optimized-fit to those targets. The comparison of objective measures like aided threshold, speech intelligibility index (SII) or articulation index (AI) and word recognition in quiet between hearing aids fitted with first-fit and optimized-fit to NAL-NL1 target is sparse in literature compared to the studies on coupler measures. It is also important to note that when compared to NAL-NL2, the NAL-NL1 prescribes high gain with lower compression ratios for adults to maximize the speech intelligibility^(18)^. It is important to explore the difference in aided performance with NAL-NL1 first-fit and optimized-fit to NAL-NL1 as it is one of the commonly used prescriptive targets with the adult population. Therefore, the current study aimed to investigate the aided performance with manufacturer NAL-NL1 first-fit and optimized-fit to NAL-NL1 target.

### Objectives of the study

To compare the real-ear aided response (REAR), the real ear insertion gain (REIG), aided thresholds, articulation index (AI) and the word recognition score (WRS) in quiet obtained with hearing aid set at the manufacturer’s NAL-NL1 first-fit and the optimized-fit to NAL-NL1 target using probe-microphone measurement.

## METHODS

### Study design

The objectives of the study were addressed through a cross-sectional within group repeated-measure experimental design.

### Participants

The study was carried out in 11 native Kannada speaking adults, in the age range from 23 to 55 years (mean age = 41.09±9.95). This sample size was found to be adequate in G*Power analysis using F-family of tests set to 0.80 power at an error rate of 5% for detecting the mean difference in aided threshold and word recognition between hearing aid fittings with a large effect size as demonstrated in this study.

The inclusion criteria include (a) bilateral sensorineural hearing loss with either flat or sloping audiogram configuration, (b) pure-tone average (PTA) between 40 and 70 dB HL, (c) greater than 60% speech identification score in quiet, (d) naïve hearing aid user, (e) ‘A’ type tympanogram with reflex present at least in one frequency, (f) post-lingual onset of hearing loss. Those having any history or presence of middle ear infection, neurological (including auditory neuropathy and/or retrocochlear pathology) or psychological complaints were excluded. The experiments were carried out in the right ear if the hearing loss was symmetrical; and only in the better ear of the participants in case the hearing loss was asymmetrical. The demographic details of the participants were given in Table 1. The mean four frequencies PTA for the test ears was 51.59±10.56 dB HL. The mean speech identification score for the test ears was 91.45±7.9%.

**Table 1:** Demographic details of the participants

**Participants**	**Gender**	**Age**	**Test Ear**	**Test Ear PTA in dB HL**	**Test Ear WRS in percentage**	**Configuration**
S1	Male	31	Right	46.25	100	Flat
S2	Male	41	Right	47.5	92	Flat
S3	Male	41	Right	50	96	Flat
S4	Male	42	Right	45	100	Flat
S5	Male	23	Right	62.5	92	Sloping
S6	Male	32	Right	31.25	100	Sloping
S7	Male	49	Right	68.75	76	Sloping
S8	Female	51	Right	47.5	88	Sloping
S9	Female	51	Right	65	90	Sloping
S10	Male	55	Right	51.25	80	Sloping
S11	Male	36	Left	52.5	92	Sloping

*Note:* PTA: Four frequency Pure-tone average; WRS: Word Recognition Score; dB HL: Decibel hearing level

### Procedures

All procedures carried out in this study adhered to the institutional ethical guidelines for bio-behavioural research on human subjects. The study protocol was also approved by the ethical committee of the institute (ECC-Res.art/03/2020). Informed consent was obtained from the participants prior to the experiments. All testing was carried out in a single- or double- sound treated room situation having ambient noise levels within the permissible limits (ref: ANSI S3.1-1999, R2018)^(19)^. Otoscopy was performed to ensure that there was no contraindication for hearing aid testing. Pure-tone audiometry was carried out using modified Hughson-Westlake procedure^(20)^ to establish the air-conduction and bone-conduction thresholds for octave frequencies from 250 Hz to 8000 Hz and 250 Hz to 4000 Hz respectively. Speech audiometry was carried out to obtain Speech Recognition Threshold (SRT) using the Kannada paired-word list^(21)^ and Word Recognition Score (WRS) at 40 dBSL (re: SRT) using phonemically balanced Kannada word identification test for adults^(22)^. Immittance audiometry was carried out using 226 Hz probe-tone at 85 dB SPL to rule out the middle ear pathology.

A 16-channel non-linear digital behind the ear (BTE) hearing aid with a fitting range of mild to severe hearing loss was used for aided testing. The hearing aid was coupled to the test ear with appropriately sized ear-tip. A personal computer with NOAH and the hearing aid specific software installed was used for hearing aid programming. HiPro was used as the interface between the hearing aid and programming computer. The participant’s details and audiogram were entered in the NOAH software. Initially, the hearing aid was programmed for first-fit with the NAL-NL1 prescriptive formula. The acclimatization level was set as ‘2’ as the participants were naïve users of hearing aids. The compression parameters were set according to the NAL-NL1 prescription. The other features like noise reduction, directional microphone was switched off to avoid the influence of these on speech identification testing. Then, the probe-microphone measures, like REAR and REIG, for this setting were carried out. The protocol for probe-microphone measurement is given in Table 2.

**Table 2:** The test protocol for probe microphone measurements

Parameter	Setting
REUR auto-adjust	On
Reference mic	On
Reference mic position	Above the pinna of the test ear
Location of probe tube mic set	Ear canal of the test ear
Sound field	12 inch from the participant’s head at 45 degree Azimuth (between the nose and the test ear)
Projected noise reduction	8x
Fitting rule	NAL-NL1
Aid type	AGC
Aid limit	Multichannel
Number of channels	16
Fit type	Unilateral
Stimulus	ANSI Digi-speech
Stimulus level	60 dB SPL

*Note:* REUR: Real-ear unaided response; NAL-NL1: National Acoustic Laboratory-Non-Linear version1; AGC: Automatic gain control; ANSI: American National Standards Institute; dB SPL: Decibel sound pressure level

Each participant was instructed to sit comfortably with their head held straight forward during the measurements. The participant’s audiogram details were entered in hearing aid test system. The positioning of the probe-tube was made using visually assisted positioning method. A marking was made on the probe-tube at 30 mm from the probe-tube tip. The probe-tube was inserted into the ear canal with the marking on the probe-tube located at the intertragal notch. Thus, it was ensured that the tip of the tube was placed 5-6 mm farther to the tympanic membrane. Then, the real-ear measurement system was leveled to ensure that the input to the hearing aid was controlled across frequency. Followed by leveling, the real-ear unaided response (REUR) was obtained at 60 dB SPL. The hearing aid and ear-tip were inserted into the ear canal without disturbing the probe tube. The REAR and resulting REIG were measured at 60 dB SPL. These probe-microphone measurements were made with the manufacturer NAL-NL1 first-fit. This program was saved as ‘Program 1’ in the hearing aid which was the NAL-NL1 first-fit program.

A second program ‘Program 2’ which was a duplicate of the ‘Program 1’ was created in the same hearing aid. The ‘Program 2’ was further programmed to match with the NAL-NL1 targets during verification. The frequency-gain adjustments were carried out until REIG closely matched to NAL-NL1 target at 60 dB SPL input level. The REAR and REIG data were recorded for the ‘Program 2’ which was the optimized-fit to NAL-NL1 target. The Program 1 (Aided 1 condition) and Program 2 (Aided 2 condition) were used to collect data on aided thresholds, AI, and aided WRS. The aided 1 and aided 2 conditions were randomized across participants to avoid the order effect.

The calibrated clinical audiometer was used to assess the unaided and aided performance in sound-field where the loudspeaker was placed 1 meter from the participant at 0^0^ Azimuth. The unaided and the aided thresholds for the initial-fit (Aided 1) and optimized-fit (Aided 2) for octave frequencies from 250 Hz to 4000 Hz were obtained using warble tones. The aided thresholds were then plotted on AI audiogram given by Humes ^[23]^ which represents a speech frequency importance function weighted AI. The AI ranges from 0 to 1 or 0.99. The audiogram has a total of 33 dots covering the speech dynamic range and each dot in the audiogram contributes 0.03 times to the AI. The total number of dots below the aided threshold curve was counted and multiplied by 0.03 to get the AI for both aided conditions.

The unaided and aided WRS for the two aided conditions were obtained using phonemically balanced Kannada word identification test for adults^(22)^. It consisted of four lists of 25 words each. The word lists were presented at 45 dB HL. It was made sure that the word lists were randomly presented across participants and test conditions. Each correct response was given a score of one and incorrect response was scored zero. The maximum word recognition score was 25.

The obtained data were analyzed using Statistical Package for the Social Sciences Version 20 software (IBM corp., Armonk, NY, USA). Descriptive statistics was carried out to obtain the mean and standard deviation for REAR, REIG, aided thresholds, AI and WRS. The Kolmogorov-Smirnov Z-test of normality revealed that the data were in normal distribution (p>0.05), thus parametric inferential statistics was carried out. The effect size was reported using partial eta squared (η_p_^2^) and Cohen’s d for ANOVA and paired t-test respectively.

## RESULTS

### Real-ear aided response and real-ear insertion gain

**Figure 1:**
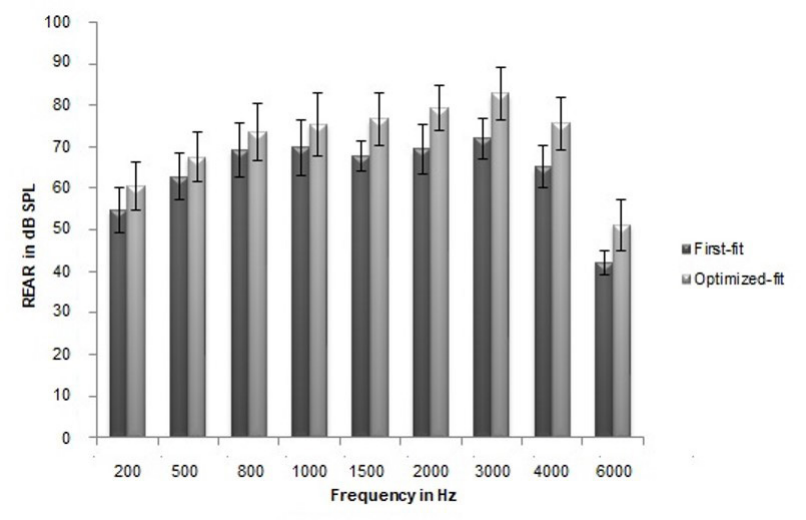
Mean of REAR (in dB SPL) across frequency for NAL-NL1 target for moderate levels (NAL-NL1 first-fit and optimized-fit) (n=11). Error bars indicate one standard deviation

**Figure 2:**
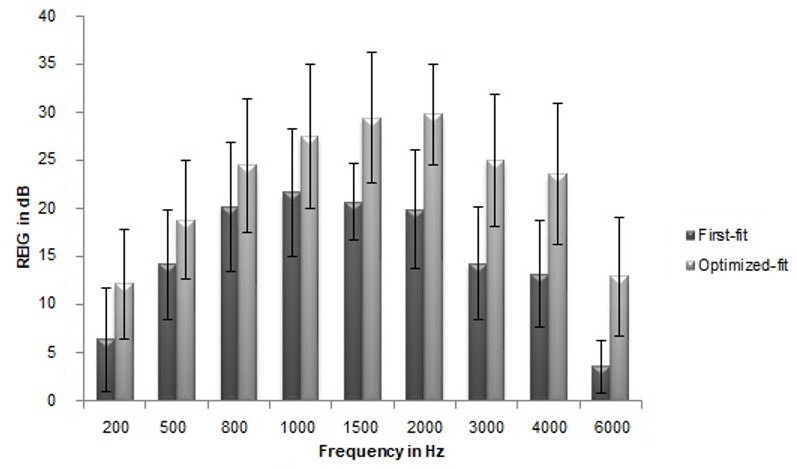
Mean of REIG (in dB) across frequency for moderate levels (NAL-NL1 first-fit and optimized-fit) (n=11), for NAL-NL1 target. Error bars indicate one standard deviation

The mean REAR and REIG measured with first-fit across frequencies were found to be less than the optimized-fit to the NAL-NL1 targets. The mean REIG data across frequencies revealed that the gain at the high frequency was much compromised in manufacturer’s first-fit than the optimized-fit to NAL-NL1 target. Figure 1 and 2 depict the mean and SD of REAR and REIG across frequencies from 200 Hz to 6000 Hz in octaves and mid-octaves.

The result of repeated measures ANOVA revealed that this difference between the first-fit and optimized-fit was statistically significant for both REAR [F (1, 10) = 207.364, p = 0.000] and REIG [F (1, 10) = 192.752, p < 0.001], with an effect size of partial eta squared (η_p_^2^) equaling 0.954 and 0.951 respectively. Pair-wise comparison with the Bonferroni adjustment also indicated that the mean difference in the REAR and REIG between the two aided conditions was significant for all frequencies considered (p < 0.005).

### Aided thresholds and aided articulation index

**Figure 3:**
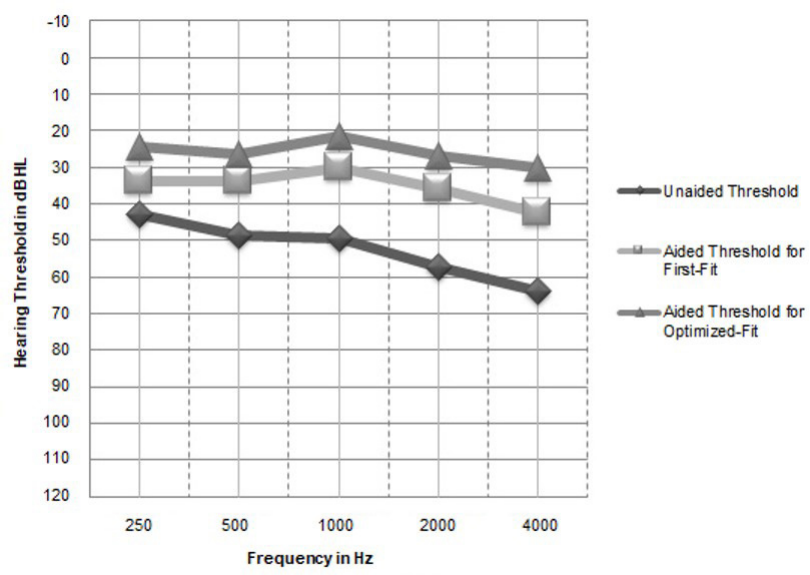
The mean unaided thresholds and aided thresholds (in dB HL) for manufacturer NAL-NL1 first-fit and optimized-fit (n=11)

The mean unaided thresholds and the aided thresholds in Aided 1 and Aided 2 test conditions are illustrated in Figure 3. It was evident that the aided benefit with the manufacturer’s first-fit was up to 10 to 15 dB HL for the low frequencies and 20 dB HL for the high frequencies. It was also observed that there was a mean aided advantage of 20 dB HL for the low frequencies and 30 dB HL for the high frequencies with the optimized-fit. The aided thresholds with optimized-fit across frequencies were improved by an average of 10 dB when compared to the manufacturer’s first-fit.

The repeated measures ANOVA was carried out with aided threshold dependent variable and the test condition (first-fit and optimized-fit) as independent variable. The results revealed a significant difference in aided threshold between the two test conditions [F (1, 0) = 51.916, p < 0.001, η_p_^2^ = 0.838]. Pair-wise comparison with the Bonferroni adjustment also indicated that there was a statistically significant aided advantage with optimized-fit than the first-fit at all frequencies (p < 0.005).

The mean and SD for AI calculated for the aided condition is given in Table 3. The paired t-test was done to compare the mean AI calculated for first-fit and optimized-fit. The results revealed that the mean AI measured for optimized-fit is statistically better than the first-fit [t (10) = 6.897, p < 0.001, with effect size Cohen’s d = 2.079].

**Table 3:** Mean and standard deviation (SD) of articulation index for two aided conditions

*Test Conditions*	*n*	*AI*	
		*Mean*	*SD*
Aided 1: First-fit	11	0.4173	0.25
Aided 2: Optimized-fit	11	0.7009	0.20

*Note:* n: Number of participants, AI: Articulation index, SD: Standard deviation

### Aided word recognition score

The Table 4 shows the mean and SD of WRS for the unaided and aided conditions. There was an aided improvement of 8% with manufacturer first-fit and 15% improvement with optimized-fit to NAL-NL1. The optimized-fit provided an average 7% improvement when compared with the NAL-NL1 first-fit.

**Table 4:** Mean and standard deviation (SD) of word recognition score (Max. score 25) for unaided condition and two aided conditions (n=11)

*Test Conditions*	*WRS*	
	*Mean*	*SD*
Unaided	19.64	3.29
Aided 1: First-fit	21.82	1.78
Aided 2: Optimized-fit	23.55	1.21

*Note:* WRS: Word recognition score, SD: Standard deviation

Paired t-test was carried out to analyse whether the mean difference between the two aided conditions was statistically significant. It indicated that the mean aided word recognition with optimized-fit to NAL-NL1 was significantly better than the NAL-NL1 first-fit [t (10) = 3.413, p = 0.007, with effect size, Cohen’s d = 1.030].

## DISCUSSION

The primary objective of the study was to compare the REAR, REIG, aided threshold, articulation index and word recognition score in two aided conditions, i.e., with the manufacturer NAL-NL1 first-fit and optimized-fit to NAL-NL1. There was a substantial difference in the measured REAR and the REIG between the manufacturer NAL-NL1 first-fit and optimized-fit to NAL-NL1 target which was more pronounced for the high frequencies. The findings of previous studies have also showed that the gain/output provided by the manufacturer’s first-fit will be significantly lesser than the generic prescriptive targets when measured both in 2 cc coupler and probe-microphone approach^(8-10)^. The difference in the individual ear canal acoustic characteristics would attribute to the reduction in gain measured with manufacturer first-fit setting^(10)^. This significant reduction in gain especially in high frequency region with the first-fit would lead to a disadvantage in the audibility and speech recognition.

The current findings showed that the audibility for soft sounds was improved with optimized-fit by 10 dB HL as revealed by the aided thresholds. There was more advantage in the high frequency with optimized-fit than the first-fit. Previous research suggests that a first-fit reduces the audibility of high-frequency signals, which could negatively impact speech recognition^(11-16)^. In the present study, the high frequency advantage in the audibility with the optimized-fit was reflected in the advantage seen in the word recognition in quiet.

Sanders et al.^(15)^ depicted that the derived speech intelligibility index (SII) from manufacturer’s default fitting was significantly lower than that of the programmed-fit to NAL-NL2 targets. They reported that the average SII obtained for moderate level sound with default fitting ranged from 0.46 to 0.56 compared to 0.65 for the NAL-NL2 fitting. Similar line of findings was observed with the AI in this study suggesting that the manufacturer setting will compromise the speech audibility and speech recognition.

The results on the word recognition revealed a mean programmed-fit advantage of 15% for a 60 dB SPL input level. This agrees with the findings reported by Valente et al.^(17)^ This was because of the improvement in insertion gain at high frequencies as obtained in probe-microphone measurement for the programmed-fit. The gain for the high frequency was increased to 10 to 12 dB from the NAL-NL1 first-fit to match with the NAL-NL1 targets. It is well documented that the high frequency information is important for perception of majority of the consonants. The high frequency advantage with the programmed-fit resulted in improvement in word recognition score.

Though the current study tried to highlight the necessity of routine use of probe-microphone measurement in hearing aid fitting, the limitations in this research need to be discussed. Aided thresholds were used as one of the verification measures. Though aided threshold can draw the information on the aided audibility for soft level sound^(23)^, it is very rarely used as a verification tool in hearing aid fitting because of various limitations pertaining to it. A major drawback of aided threshold is the inability to verifying the non-linear hearing aids. The non-linear hearing aids vary the gain depending on the input level. Also, to obtain the true aided thresholds, the sound field audiometer must be calibrated, the patients must be seated with minimal head movements, and the involvement of the non-test ear must be eliminated. In the present study it was ensured that all these factors were controlled. The audiometer was calibrated, participants were instructed to maintain the position of the head throughout the testing, and the better ear was the test ear whenever there was an asymmetrical hearing loss. Though caution is to be exercised while interpreting the aided thresholds, it is one of the commonly used verification method for implantable hearing devices such as bone anchored hearing devices, middle ear implants and cochlear implants, where probe-microphone measures cannot be used as verification option^(23)^.

The recognition of speech cannot be directly inferred from the aided thresholds^(24)^. However, the aided thresholds can be utilized for calculating the articulation index (AI) or speech intelligibility index (SII) and thereby predict the speech recognition^(25-27)^. This is based on a same underline theory that speech recognition is related to the audible speech sounds. In this study, the AI was used utilizing the count-the-dot audiogram^(28)^ which is considered as one of the easiest clinically feasible method to estimate AI. AI was calculated from hearing threshold of the listener and the long-term average speech spectrum reaching the listener’s ear^(26)^.

Modifications were made into the calculation of AI to make it more effective for predicting the speech recognition and it was renamed as SII in ANSI S3.5‐1997 standards^(27)^. The SII calculation incorporated updated technical information on spread of masking, standard speech spectrum level, relative importance of various frequencies to speech intelligibility, speech level distortion, and hearing loss desensitization. Thus, make the SII to predict speech recognition appropriate for many conditions of use where the traditional AI does not apply. These procedures are automatized and incorporated in commercially available probe microphone equipment. The display provides SII for different speech level like soft (55 dB SPL), average (65 dB SPL), and loud levels (75 dB SPL) which enables the audiologist to counsel the patient on the effect of hearing loss on unaided and aided speech recognition different input levels.

This study was carried out using a single hearing aid model of a manufacturer. It is important to gather more evidence with other hearing aid makes and their default first-fit settings. Based on other reports showing the disadvantage of high frequency gain with manufacturer’s defaults with a variety of hearing aid models^(10,14)^, it can be assumed that other hearing aids models would also exhibit poor aided performance with manufacturer’s first-fit program. It is also important to evaluate perceptual effect of first-fit and optimized-fit with the other available generic formulae in different population, especially in pediatric population.

The participants recruited for the study were with moderate and moderately severe SNHL. It will be interesting if future research of same kind would target on different degree and configuration of hearing loss. Aarts and Caffee^(11)^ found that the measured REAR values were significantly different from the manufacturer predicted initial-fit REAR values in 41 participants with two audiogram configurations using two different input levels (50 and 90 dB SPL). This difference would also influence the aided response and subjective satisfaction in subjects with different degree and configuration of hearing loss which need to be investigated further. In addition, the long-term outcome or acclimatization and real-world benefit with given fittings need to be investigated.

As a health care professional, it is the responsibility of the audiologists to make their clients satisfied with the hearing aid fitting and to encourage them to use the hearing aid regularly. It will further prevent the auditory deprivation, cognitive decline and social isolation associated with hearing loss. A standard hearing aid fitting protocol including the probe-microphone measurement can improve the patient’s self-perceived benefit of their hearing aids, contentment with the hearing care professional and result in better patient loyalty. It was reported that inclusion of probe-microphone measurement in clinical protocol positively influences the user’s trust and improve the acceptance of hearing aid and its use^(29,30)^.

To conclude, the result of the current study also revealed improvement in aided threshold, articulation index and word recognition when the verification of gain and output was carried out through probe microphone measurement. The probe-microphone measurement is considered as the “Gold Standard” for verification of hearing aid fitting in the best practice guidelines for hearing aid fitting. Many audiologists are not using the probe-microphone technique regularly to verify the hearing aid gain and output when dispensing it. It is depicted that lack of verification will result in underamplification in the higher frequencies, that in turn cause inaudibility for soft and average level sounds and this in turn would compromise the speech recognition. A large number of hearing aid users are now using unverified fit that may be one of the reasons for rejection of amplification and dissatisfaction in adult population. Thus, it can be inferred that the clinical use of probe-microphone measurement is warranted to yield better audibility and speech recognition and in turn better satisfaction and quality of life.
